# Effects of lifestyle-related risk factors on life expectancy: A comprehensive model for use in early prevention of premature mortality from noncommunicable diseases

**DOI:** 10.1371/journal.pone.0298696

**Published:** 2024-03-14

**Authors:** Beata Jackowska, Piotr Wiśniewski, Tomasz Noiński, Piotr Bandosz

**Affiliations:** 1 Department of Statistics, Faculty of Management, University of Gdańsk, Sopot, Poland; 2 Chair and Department of Endocrinology and Internal Diseases, Medical University of Gdańsk, Gdańsk, Poland; 3 Lab4Life Sp. z o.o., Gdynia, Poland; 4 Division of Preventive Medicine & Education, Medical University of Gdańsk, Gdańsk, Poland; University of Leeds, UNITED KINGDOM

## Abstract

Morbidity and premature mortality from noncommunicable diseases can be largely prevented by adopting a healthy lifestyle at the earliest possible age. However, tools designed for the early identification of those at risk among young adults are lacking. We developed and validated a multivariable model for the prediction of life expectancy, allowing the early identification of apparently healthy adults at risk of lifestyle-related diseases. We used a cross-sectional approach to calculate life expectancy using data from 38,481 participants of the National Health and Nutrition Examination Survey (1999–2014), aged ≥20 years. A multivariable logistic model was used to quantify the impact of risk factors on mortality. The model included the following lifestyle-related mortality risk factors as predictors: smoking, diet, physical activity, and body mass index. The presence of the following chronic diseases was considered: diabetes, arrhythmia, coronary artery disease, myocardial infarction, stroke, and malignant neoplasms. The model showed a good predictive ability; the area under the receiver operating characteristic curve measure was 0.846 (95% uncertainty interval 0.838–0.859). Life expectancy was determined using the life table method and the period life tables for the US population as the baseline. The results of this model underscore the importance of lifestyle-related risk factors in life expectancy. The difference between life expectancy for 30-year-old individuals with lifestyle characteristics ranked in 90% and 10% of their gender and age groups was 23 years for males and 18 years for females, whereas in 75% and 25%, it was 14 years for males and 10 years for females. In addition to early risk identification, the model estimates the deferred effect of lifestyle and the impact of lifestyle changes on life expectancy. Thus, it can be used in early prevention to demonstrate the potential risks and benefits of complex lifestyle modifications for educational purposes or to motivate behavioral changes.

## Introduction

Noncommunicable diseases (NCDs), such as cardiovascular diseases, cancer, diabetes, and chronic respiratory diseases, are the leading cause of death and disability worldwide [1]. Morbidity and premature mortality due to NCDs can be avoided by adopting healthy lifestyles. Early prevention strategies aimed at modifying adverse levels of risk factors (i.e., primary prevention) or preventing the development of risk factors (i.e., primordial prevention) are of particular importance [2]. They are primarily based on the identification of healthy individuals at risk, periodic assessment of risk factors, and lifestyle optimization. They are intended to begin at an early age and continue through a lifetime [2–4].

Several epidemiological studies have evaluated the effects of lifestyle-related risk factors on health outcomes and have consistently suggested the importance of healthy lifestyle behaviors for the prevention of diseases and premature mortality [5–10]. However, these studies were mostly conducted on middle-aged and older adults. These conclusions may not be directly extrapolated to young adults who differ in terms of lifestyle and prevalence of risk factors. In some studies, lifestyle risk factors were represented jointly by one variable (i.e., the number of adverse risk factors) [5, 9, 10], while others lacked diet quality [7, 8] or physical activity [7]. Most studies [7–10] categorized lifestyle-related predictors into a small number of broad categories (e.g., current smoker/former smoker/never smoked). This may lead to a loss of information on the intensity of the predictor, decrease the amount of variation in the data, and raise concerns about the value of the cutoffs used.

Several clinical scales have been developed to identify high-risk individuals, including the SCORE2 [11], SCORE-OP [12], Framingham [13], and Q-risk [14]. However, they are intended for middle-aged and older populations, are limited to cardiovascular outcomes, and assess only short-term risks. These predictions are based on variables that are a consequence of lifestyle rather than its features (for example, hypertension or hypercholestrolemia).

This study aimed to develop a model that overcomes the above issues and is suitable for the early identification of apparently healthy adults at risk of lifestyle-related diseases using life expectancy (LE) as a general measure of health conditions.

## Materials and methods

### Overall design

A multivariable logistic model was used to identify risk factors for mortality and estimate their relative effects in terms of odds ratios (ORs). To calculate individual LE (i.e., LE for individuals with specified values of covariates), we used the life table method to estimate the “personalized” period (current) life table. We addressed the short-term horizon of exposure to death risk by replacing long-term cohort studies with a cross-sectional approach in which all analyzed age groups observed in the short period formed a hypothetical (synthetic) cohort. This approach is commonly used in demographic analyses as opposed to the life table for a real cohort, cf. e.g. [15, 16]. We applied the population period life table and population characteristics as the baseline. Our model estimates LE using a “personalized” period life table in which the conditional probabilities of death are converted to odds and adjusted with the odds ratios (ORs) obtained from a multivariable logistic regression model of short-term mortality.

### Study population

We used data from the 1999–2014 cycles of the National Health and Nutrition Examination Survey (NHANES). The NHANES is a cyclical survey representative of the U.S. civilian, non-institutionalized population. Details of its design and procedures are available at the Center for Disease Control and Prevention (CDC) website [17]. Briefly, the NHANES includes cross-sectional surveys based on a stratified multistage probability sampling design with the oversampling of specific subpopulations (a complex sampling design). NHANES is designed to produce stable prevalence estimates for population subgroups (domains) defined by age group, sex, low-income status, among others. Oversampling is done to increase the reliability and precision of estimates of health status indicators for these population subgroups, including the elderly. Sample weights allow estimates from these subgroups to be combined to obtain national estimates that reflect the relative proportions of these groups in the population as a whole [18]. Each survey includes two components: a household interview and health examination. Household interviews include questionnaires regarding demographic, socioeconomic, dietary, and health-related information. The health examination component consists of medical, dental, and physiological measurements as well as laboratory tests administered by trained medical personnel in a fully equipped mobile examination center.

To model short-term mortality based on risk factors, we used the 2015 Public-Use Linked Mortality Files developed by the National Center for Health Statistics (NCHS). In this dataset, NHANES data were linked to death certificate records from the National Death Index (NDI) using a probabilistic matching algorithm, with participant follow-up through December 31, 2015. The linkage methodology has been described in [19].

The NHANES study protocol as well as the linkage to the NDI was approved by the NCHS Institutional Review Board (IRB). Since the current analysis used only the deidentified public-use datasets, devoid of all direct identifiers as well as any characteristics that could lead to identification, it was not subject to IRB review.

**Fig 1:**
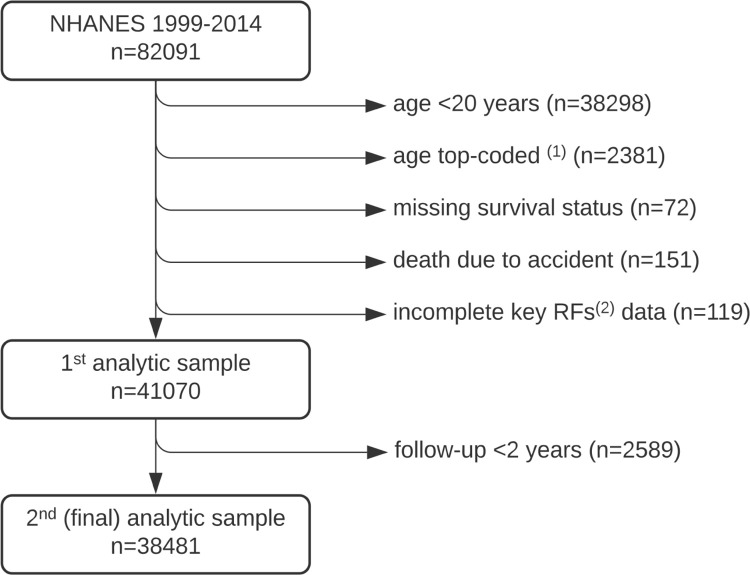
Flow diagram for the enrollment of study participants. (1) Open-ended age intervals: ≥85 years in NHANES 1999–2006 (n = 802) and ≥80 years in NHANES 2007–2014 (n = 1579); (2) RFs—qualitative risk factors.

The participant selection process and reasons for exclusion are shown in Fig 1. The initial analytical sample included 41,070 participants with a median follow-up of 98 months (maximum, 201 months). A total of 4,704 deaths were recorded during the follow-up period, including 380 deaths in the first year and 425 in the second year of the participants’ follow-up since the survey. During the short-term mortality modeling, due to the number of predictors, the one-year follow-up had to be extended into a two-year period which resulted in an exclusion of 2,589 additional participants alive on December 31, 2015 due to shorter follow-up period.

Data from the Human Mortality Database (HMD) [20] for the years 1999–2014 in the US population were used to estimate sex- and age-specific death probabilities. HMD collates mortality and population data from authoritative national agencies subject to a uniform set of procedures, accounting for various kinds of data idiosyncrasies and disruptions; uniform methods ensure high consistency of the output data across time and countries [21].

### Risk factors for mortality

Predictors were prespecified based on expert discussion supported by a recently published systematic review of the literature with a meta-analysis of 74 prospective cohort studies (2.5 mln participants) investigating the association between lifestyle factors and all-cause mortality [22], and the availability of data on the study participants. We avoided predictors that could be treated as lifestyle mediators, and potentially could take over part of the effect of lifestyle-related variables in the regression model. For example, the total cholesterol level, a strong and causal risk factor for CVD mortality, is causally related to diet quality. We designed our model to evaluate the effects of dietary changes rather than direct changes in cholesterol (i.e., pharmacotherapy). Similarly, we did not include blood pressure, but rather related lifestyle variables such as physical activity and BMI. Our basic set of predictors included age, gender, BMI, physical activity, diet quality, and cigarette smoking.

Smoking exposure was defined as the cumulative number of cigarette packs smoked over a lifetime (in thousands of packs). To model the decrease in risk after smoking cessation, we assumed a reduction rate of 1/25 of the lifetime smoking exposure per year, which resulted from the interaction between time since quitting smoking and cumulative number of cigarettes as risk factors for death in the analyzed NHANES dataset. Thus, the negative effects of smoking disappeared after 25 years. Physical activity was derived from the NHANES questionnaires as the sum of leisure-time moderate or vigorous activity, measured in MET minutes per week (MET: metabolic equivalent of task). Diet quality was assessed using a proprietary score inspired by the Mediterranean diet [23]. The score was calculated using 22 dietary-related variables in the NHANES 24-hour recalls and included general eating habits and diet content (eating breakfast, eating multiple meals a day, homemade meals, snacking, type of snacks, sweeteners, servings of fruits, vegetables, whole grains, legumes, nuts, dairy products, amount and type of bread, amount and type of meat, fish, sweets, mono- and polyunsaturated fats, drinking at least 1500 mL of water, consumption of soft drinks, and alcohol). Higher scores indicate better diet quality.

To account for the presence of chronic diseases in the estimation of LE, we included a history of the following diseases as covariates: diabetes, coronary heart disease, stroke, irregular pulse on physical examination (as a proxy for atrial fibrillation), and relevant cancers. We selected cancers with relatively high mortality and prevalence (i.e., cancer of the bladder, breast, colon, esophagus, kidney, larynx, primary liver, lung, pancreas, and stomach).

### Statistical analysis

#### Data preparation

Data preparation included missing data analysis together with imputation, and winsorizing of continuous predictors at the 1st and 99th percentiles. BMI was transformed using a custom function f(BMI) to maintain this variable as continuous and extend its range while preserving the U-shaped BMI-mortality relationship. The function of the BMI was defined as constant for the normal BMI range (18.5 to 25 kg/m^2^), as decreasing for very low BMI (under 18.5 kg/m^2^), and increasing for high BMI (25 kg/m^2^ and more). The variable “physical activity” was log-transformed due to the wide range of values. No multicollinearity or strong correlations were found among the predictors.

#### Quantification of the impact of risk factors on mortality

The association between the risk factors and mortality was expressed as ORs derived from a multivariable logistic model. This approach allowed the estimation of the effect of individual predictors while keeping other risk factors constant. The complex sample design of the NHANES was included in the OR estimation, which allowed for generalization of the results. The calculations were performed using the ’survey’ R package [24]. Initially, we modeled one-year all-cause mortality; however, as the number of predictors increased, we switched to a two-year period to maintain a reasonable number of events (both total and per variable). This made our study conditions satisfactory, because during estimation of the logistic model, the problem is not the frequency of rare events but the number of events and the number of events per variable [25, 26]. Given the relatively short two-year follow-up period, we assumed time-invariant covariates for the study participants.

The selection of independent variables for use in the multivariable model was performed iteratively by adding or removing variables based primarily on subject knowledge and supported by statistical techniques. In each iteration step, changes in the estimates were controlled to avoid mediators. Because the effect of physical activity on mortality was found to be too high compared to that found in the literature, we specified its regression coefficient (parameter *β*_*i*_) a priori based on literature data [27, 28] and then re-estimated the other model parameters. This large effect may be due to reverse causation and confounding factors. Although low physical activity is a causal risk factor for mortality, it may also be a marker of low physical capacity due to life-shortening diseases [29].

Two interactions were included in the model: age with gender, and age with f(BMI). As the influence of BMI on the risk of mortality decreased with age, a limiting condition was imposed on the regression coefficients for f(BMI) and the interaction between age and f(BMI); as a result, at each age under consideration, the sum of both coefficients was positive. This was aimed to alleviate the effects of the reverse causation (the "obesity paradox") in older age (in our study, this effect was found after the age of 60), i.e. to preserve the negative impact of obesity on the risk of mortality at all ages [30].

The following were used during model building: measures of the predictive power of independent variables (Information Value, Cramer’s V, Gini) for predictor selection, measures of model performance (area under the receiver operating characteristic curve (AUC), sensitivity, specificity, the Brier quadratic probability score), and p-value in the Wald significance test for regression coefficients. The Holm method was used to control for the risk of type I errors in multiple testing.

#### Assessment of baseline population characteristics

Baseline population characteristics were obtained from the NHANES survey data. The means of the quantitative predictors and prevalence of the qualitative predictors were calculated by age and gender categories and smoothed over age with splines. For the baseline probabilities of death, we took the one-year death probabilities by sex and age (*q*_*x*_) from the population life table based on the HMD. These probabilities were converted into two-year probabilities.

#### Assessment of individual exposure

To calculate an individual’s LE, individual characteristics and the presence or absence of risk factors at the time of the survey and in the subsequent years of life were assessed. Because many risk factors change with age, we estimated their expected values in the following years of life. For continuous risk factors, we simply modeled future expected values using nonlinear regression; the fourth-degree polynomial was fitted for BMI, and the exponential function was fitted for physical activity (measured in MET minutes per week). However, the mortality logistic regression model included several chronic diseases as covariates. Apart from age and sex, the risks of these diseases are also lifestyle-related, and their expected values must be known before estimating the individual risk of death in the subsequent years of life.

Because the diseases under consideration are irreversible, we assumed that if they are already diagnosed, they will persist for the rest of an individual’s life. However, in other cases, the future risks of these diseases depend on age, sex, and lifestyle-related risk factors. We created a set of simple models to calculate the risk. To calculate the probability of suffering from the disease at a specific age and sex, we multiplied the average risk of an unexposed person by the relative risk (RR) attributed to the patient’s existing risk factors. The average risk of unexposed individuals was calculated using the concept of population attributable risk fraction (PARF) [31, 32]. PARF is interpreted as an expected proportional reduction in the average risk in the population if exposure is eliminated. Thus, by knowing the PARF and average population probability of disease, we can estimate the expected risk in a theoretically unexposed population. Then, we can calculate individual risk based on the risk of the unexposed population and the RRs of all exposures included, assuming the independence of RRs. We used the values of multiple adjusted RRs from the existing literature [33, 34].

#### Determination of life expectancy

The multivariable logistic model was only used to estimate two-year ORs and not to predict death probabilities. An accurate estimation of death probabilities by age was obtained from separate life tables for the male and female populations (see subsection Study Population). The age- and sex-specific death probabilities in the population were converted to two-year odds of dying and then adjusted using the estimated ORs for risk factors relative to the baseline population characteristics in the sex and age groups. The adjusted ORs were used to construct “personalized” life tables. Finally, “personalized” LE was calculated using the life table method, that is, the average number of years of life remaining since the person’s current age. This method of constructing “personalized” life tables ensures that the LE of a person with baseline population characteristics is the same as the LE from the life table of the population.

#### Model validation

The primary goal of the mortality risk model development was to obtain the most accurate estimates of ORs for risk factors and not to predict probability or classify individuals. The multivariable logistic model was validated using the nonparametric bootstrap method to quantify the degree of overfitting [25]. We chose bootstrapping over a train/test split because the number of outcomes of interest (i.e., deaths) was low. This also helped maintain a reasonable number of events per variable (especially for categorical variables) and preserved the structure of the data from the complex sample design during model estimation. We drew 200 bootstrap replications (accounting for the complex sample design) to estimate and correct for optimism (i.e., overfitting) in performance measures such as AUC, sensitivity, specificity, and the Brier score. From these 200 bootstrap replications, we also estimated 95% uncertainty intervals (UI) for LE.

Data operations, statistical analysis and modeling were performed using the following software: Statistica, version 13.1 (TIBCO Software Inc., CA, USA) and R software version 3.6 (R Foundation for Statistical Computing, Vienna, Austria).

## Results

### Assessment of the impact of risk factors on mortality

The characteristics of the 38,481 study participants according to two-year survival (total, survivors, and deceased) are presented in Table 1. Extending the follow-up period increased the number of observed events from 380 (0.9%) deaths within one year to 805 (2.1%) deaths within two years (see the subsection Study population). Those who survived two years after the survey, in comparison to those who died, were younger (median 44 vs 65 years), had smoked fewer cigarette packs over a lifetime (median 0 vs 2.1 thousands), were more physically active in their leisure time (median 274 vs 0 MET minutes per week), and were less likely to suffer from chronic diseases (at least one disease 16.5% vs 59.0%).

**Table 1:** Characteristics of study participants based on two-year survival (NHANES 1999–2014).

	Total	Survivors	Deceased
N unweighted (%)	38,481 (100.0%)	37,676 (97.9%)	805 (2.1%)
**Quantitative predictors**	Median (interquartile range) ^a^		
Age	45 (32, 57)	44 (32, 57)	65 (54, 75)
Lifetime smoking exposure ^b^	0.00 (0.00, 2.81)	0.00 (0.00, 2.70)	2.10 (0.00, 11.09)
BMI	27.71 (24.09, 31.89)	27.71 (24.09, 31.89)	27.61 (24.21, 31.39)
Physical activity ^c^	249 (0, 1233)	274 (0, 1247)	0 (0, 69)
Diet quality score ^d^	2.10 (-1.25, 5.61)	2.10 (-1.26, 5.65)	2.19 (-0.92, 3.81)
**Qualitative predictors**	Frequency (%)		
Gender (male)	48.4%	48.3%	57.5%
History of diabetes	9.3%	9.1%	27.1%
Irregular pulse on examination	2.4%	2.3%	9.6%
History of coronary heart disease ^e^	4.7%	4.4%	23.0%
History of stroke	2.5%	2.3%	14.2%
History of cancer ^f^	2.2%	2.1%	13.5%
MVPA^g^ = 0	41.0%	40.5%	74.2%

^a^ Positional measures due to the asymmetric distribution of variables “Lifetime smoking exposure,” BMI, and “physical activity”

^b^ Lifetime smoking exposure: the synthetic variable considers thousands of smoked cigarette packs and the mitigating effect of the time since quitting smoking.

^c^ Physical activity: the synthetic variable for moderate-to-vigorous leisure-time physical activity in MET minutes per week

^d^ Diet quality score: the synthetic variable created from the set of variables related to diet

^e^ Coronary heart disease including myocardial infarction

^f^ Cancer of any of the following: bladder, breast, colon, esophagus, kidney, larynx, liver, lung, pancreas, or stomach

^g^ MVPA–moderate-to-vigorous leisure-time physical activity

The fitted multivariable logistic model (Table 2) allowed the estimation of the effect of individual predictors while holding other risk factors constant. The model included the following explanatory variables: five continuous variables (age, lifetime smoking exposure, BMI, physical activity, and diet score), six qualitative variables (gender and history of five chronic diseases), and two interactions (age with gender and age with f(BMI)).

**Table 2:** Results of the estimation of the logistic model explaining the two-year mortality risk (NHANES 1999–2014).

Predictors		Estimate of *β*_*i*_	Standard error of estimate	Adjusted Wald P-value ^a^	Odds ratio^b^ exp(*β*_*i*_)	Lower limit of 95% CI^c^	Upper limit of 95% CI^c^
	Intercept	-10.6913	1.7029	<0.0002	0.0000	0.0000	0.0006
*x*_1_	Age– 20 ^d^	0.1337	0.0285	0.0001	1.1430	1.0809	1.2086
*x*_2_	Gender (male-1, female-0)	1.4898	0.3575	0.0003	4.4362	2.2015	8.9393
*x*_3_	Lifetime smoking exposure ^e^	0.0261	0.0047	<0.0001	1.0264	1.0170	1.0359
*x*_4_	f(BMI) ^f^	0.0660	0.0299	0.0291	1.0682	1.0075	1.1326
*x*_5_	f(Physical activity) ^g^	-0.1000	*β*_*i*_ specified a priori^h^		0.9048	OR specified a priori^h^	
*x*_6_	Diet quality score ^i^	-0.0234	0.0079	0.0119	0.9769	0.9618	0.9922
*x*_7_	History of diabetes (yes-1, no-0)	0.4526	0.1036	0.0002	1.5723	1.2834	1.9263
*x*_8_	Irregular pulse on examinations (yes-1, no-0)	0.4581	0.1826	0.0271	1.5811	1.1054	2.2615
*x*_9_	History of coronary heart disease ^j^ (yes-1, no-0)	0.6094	0.1279	<0.0001	1.8394	1.4315	2.3635
*x*_10_	History of stroke (yes-1, no-0)	0.7654	0.1247	<0.0001	2.1500	1.6839	2.7450
*x*_11_	History of cancer ^k^ (yes-1, no-0)	1.1049	0.1897	<0.0001	3.0188	2.0812	4.3787
*x*_1_ * *x*_2_	Gender*(Age– 20)	-0.0240	0.0070	0.0036	0.9763	0.9629	0.9898
*x*_1_ *x_4_	f(BMI)*(Age– 20)	-0.0011	0.0005	0.0291	0.9989	0.9979	0.9999

P-value in the Wald test for the set of predictors: p < 0.0001

^a^ The Holm method to adjust P-value for multiple testing

^b^ Odds ratio for the non-product term, ratio of the odds ratios for the product (interaction) term

^c^ 95% confidence interval (95% CI) for the odds ratio and the ratio of the odds ratios

^d^ Subtracting 20 years from age makes the youngest group in the study the reference group

^e^ Lifetime smoking exposure: the synthetic variable considers thousands of smoked cigarette packs and the mitigating effect of the time since quitting smoking

^f^ f(BMI): the function of BMI was defined as constant for the normal BMI range, as decreasing for a very low BMI, and as increasing for a high BMI

^g^ f(Physical activity): logarithmic transformation of the synthetic variable “physical activity,” i.e., moderate-to-vigorous leisure-time physical activity in MET minutes per week

^h^ The parameter *β*_*i*_ specified a priori on the basis of literature and included during model fitting

^i^ Diet quality score: the synthetic variable created from the set of variables related to diet

^j^ Coronary heart disease including myocardial infarction

^k^ Cancer of any of the following: bladder, breast, colon, esophagus, kidney, larynx, liver, lung, pancreas, or stomach

Each risk factor shown in Table 2 was significantly associated with mortality (at the significance level of 0.05). Table 2 also contains ORs with 95% confidence intervals (CI) for the non-product terms and the ratio of ORs for the product (interaction) terms. Since gender interacts with age, the presented OR showed the odds of death of males relative to females at the age of 20 years (the variable “Age—20” equals zero). Thus for 20-year-olds, the odds of death for men were 4.4 times higher than for women (95% CI 2.2–8.9). Among the diseases, a history of cancer (OR = 3.0, 95% CI 2.1–4.4) and stroke (OR = 2.2, 95% CI 1.7–2.7) had the greatest impact on the odds of death. However, some of the effects of this model could not be directly interpreted due to variable transformation or the synthetic character of some variables. Furthermore, because age interacted with f(BMI), the OR for age also had no direct interpretation.

Positive values of the model parameters (OR > 1) indicated that the probabilities and odds of death increase with age, the number of cigarettes smoked (element of the synthetic variable “lifetime smoking exposure”), and a high or very low BMI, and are greater for males and those suffering from chronic diseases. Negative values of the model parameters (OR < 1) indicated that the probabilities and odds of death decreased with years from quitting smoking (element of the synthetic variable “lifetime smoking exposure”), increased time of physical activity per week, and adhering to the principles of healthy eating.

The two interactions in the model can be explained as follows: (i) the interaction between age and gender: excess male mortality decreases with age, and (ii) the interaction between age and f(BMI): the influence of abnormal BMI on the risk of mortality decreases with age [35].

The performance measures of the multivariable logistic model (Table 3) indicate a good quality of the model, as evidenced by the results of bootstrap validation with 200 replications of the dataset: relatively small corrections of measures due to optimism (i.e., overfitting) and relatively narrow uncertainty intervals (UI). Primarily, the corrected AUC is 0.8463 (95% UI 0.8377–0.8593). According to the literature, an AUC between 0.8 and 0.9 indicates good [36] or excellent [37] discrimination.

**Table 3:** Performance measures of multivariable logistic model–validation using the 200 bootstrap replications.

Measure	Data set			Optimism	Corrected measure	95% uncertainty interval
	Original	Train	Test			
Area under the ROC curve (AUC)	0.8474	0.8480	0.8468	0.0012	0.8463	0.8377–0.8593
Sensitivity^a^	0.7964	0.7884	0.7857	0.0027	0.7937	0.7623–0.8137
Specificity^a^	0.7678	0.7675	0.7673	0.0002	0.7676	0.7464–0.7869
Brier score	0.0130	0.0130	0.0130	0.0000	0.0129	0.0122–0.0138

^a^ According to the maximized Youden index

### Determination of life expectancy depending on risk factors

**Fig 2:**
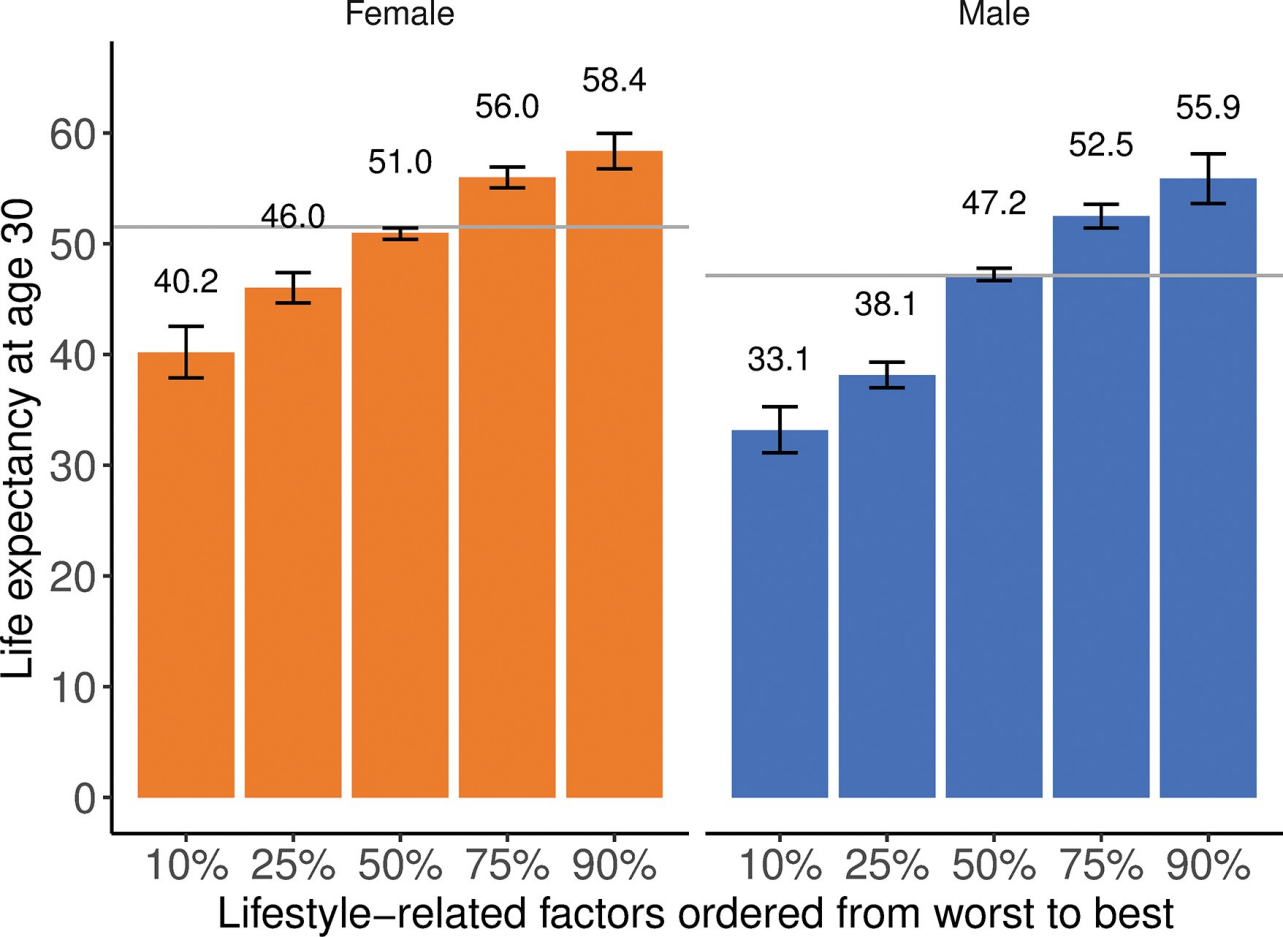
Life expectancy at age 30 (with 95% uncertainty interval) depending on lifestyle-related factors. The horizontal line marks the population LE in the sex and age group. See Table 4 for the specific values of lifestyle factors.

The use of ORs (Table 2) to correct the population life table allowed us to determine LE depending on 11 risk factors: age, gender, lifestyle factors (lifetime smoking exposure, BMI, physical activity, and diet score), and a history of five chronic diseases. Owing to the large number of predictors, we present the results for selected sets of risk factors. Fig 2 shows the results for 30-year-old men and women, with lifestyle-related factors ranked in 10%, 25%, 50%, 75%, and 90% of their age and gender groups (Table 4 presents the characteristics of these individuals). LE with a 95% UI was determined for each case.

**Table 4:** Characteristics of 30-year-old men and women in the same ranking position in their age and gender groups.

Position in ranking	Diet quality score	BMI	Physical activity^a^	History of regular cigarette smoking	History of five chronic diseases
**30-year-old men**					
10%	-16.11	35.94	0.00	Currently smoking for 15 years, 30 packs a month	None
25%	-8.64	30.70	0.00	Currently smoking for 11 years, 15 packs a month	None
50%	-1.63	27.39	120.00	Non-smoking	None
75%	13.28	24.33	420.00	Non-smoking	None
90%	25.93	21.77	778.77	Non-smoking	None
**30-year-old women**					
10%	-15.00	38.42	0.00	Currently smoking for 14 years, 15 packs a month	None
25%	-7.07	33.23	0.00	Smoking history of 8 years, 4.5 packs a month, quit 1 month ago	None
50%	-0.25	28.19	48.50	Non-smoking	None
75%	13.61	23.61	249.42	Non-smoking	None
90%	20.85	20.91	549.08	Non-smoking	None

^a^ The equivalent of leisure-time moderate activity in minutes per week

**Fig 3:**
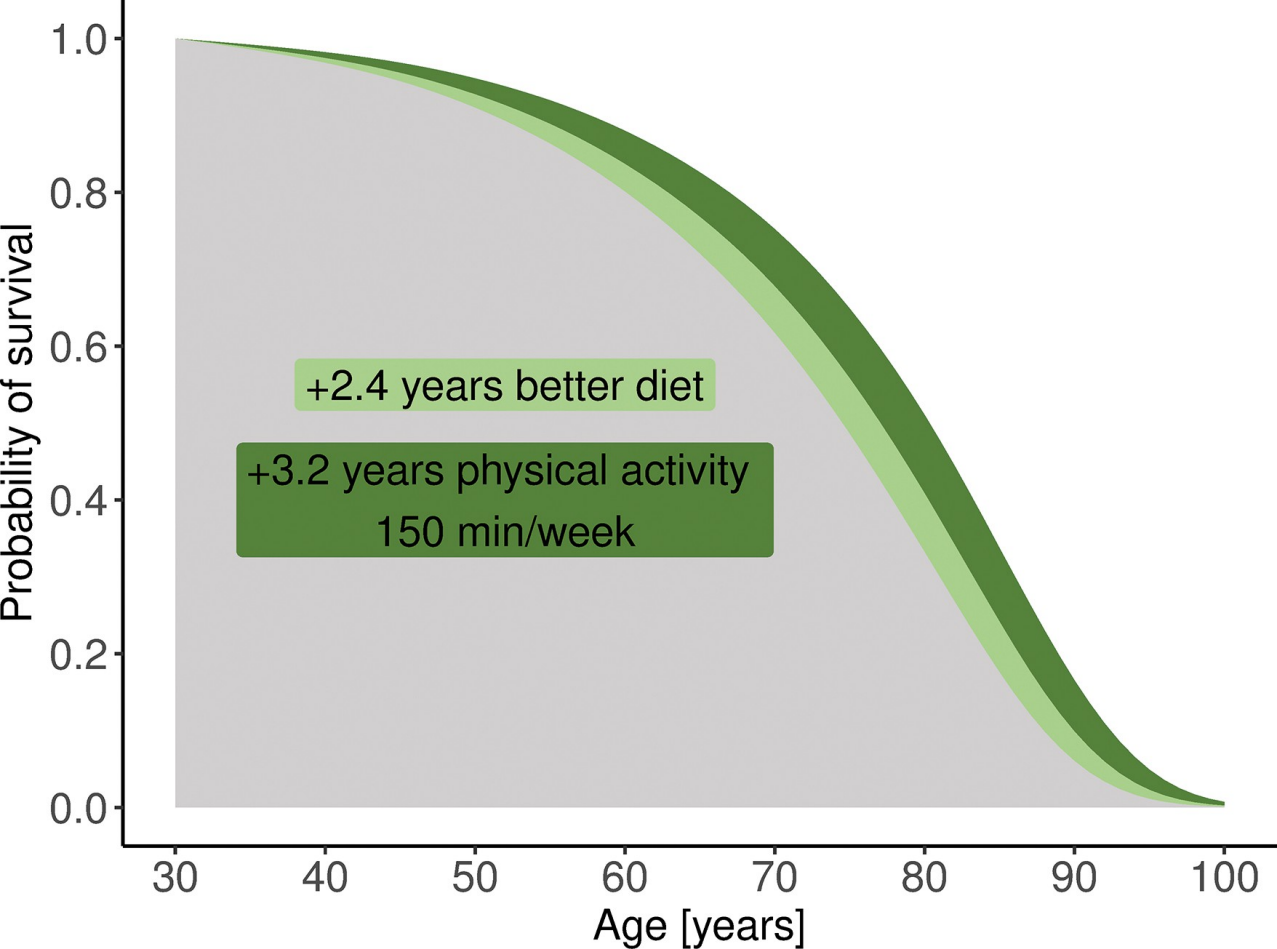
Life expectancy as the area under the survival curves–an example of a 30-year-old patient A.

**Fig 4:**
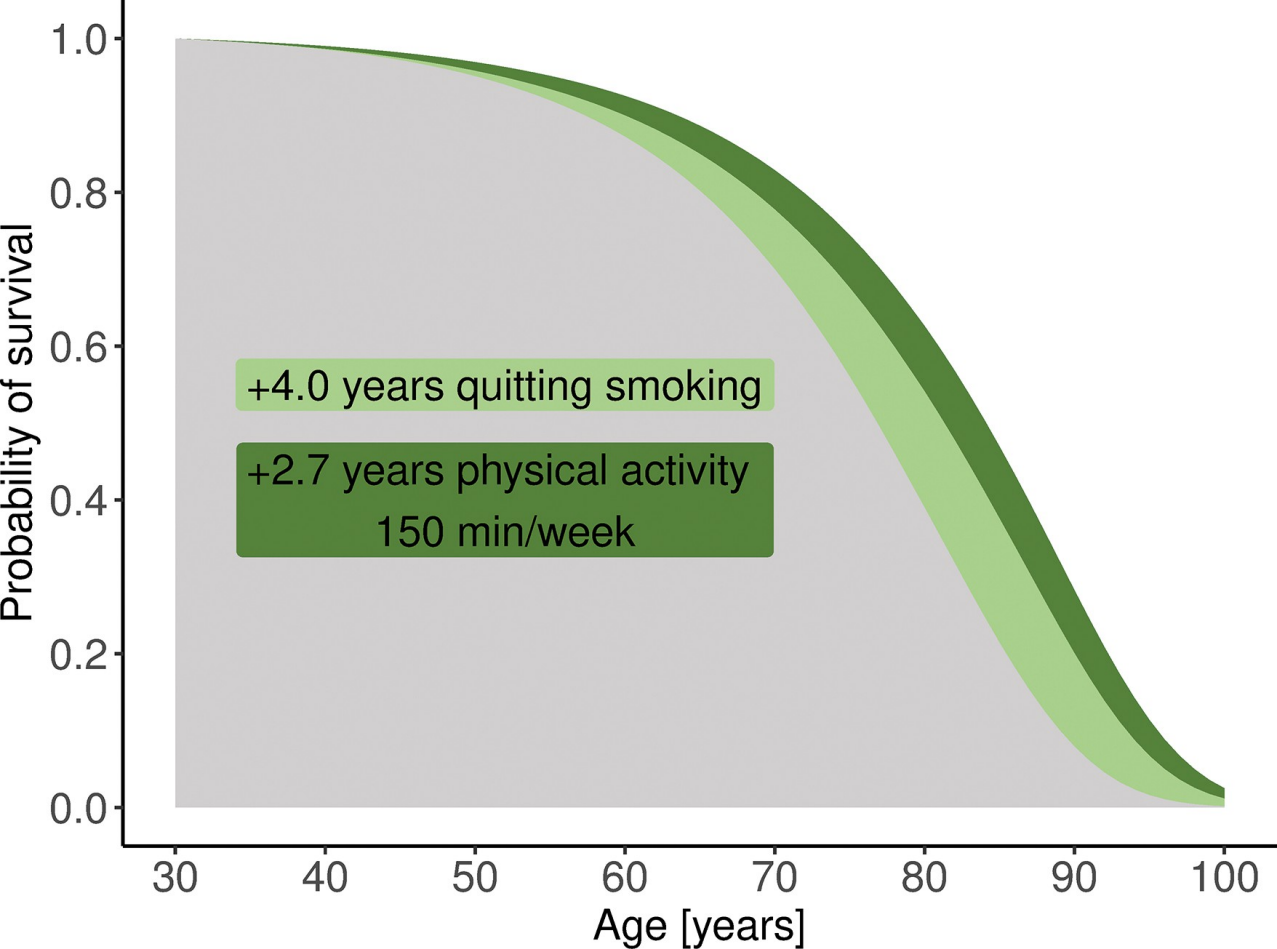
Life expectancy as the area under the survival curves–an example of the 30-year-old patient B.

Two patient examples are shown in Figs 3 and 4, in which the effects of lifestyle assessments and modifications are illustrated. The 30-year-old patient A was a healthy male distinguished by an unhealthy diet (20th percentile for diet quality in the gender and age group), low physical activity (0 min moderate-to-vigorous leisure-time physical activity), and was overweight (BMI = 28.7 kg/m^2^). His estimated LE was 41.9 years (95% UI 40.3–43.3) and was 5.3 years shorter than the population LE in the gender and age group and 20.5 years shorter than the 90th percentile for this group. Increasing leisure-time physical activity per week from 0 to 150 min of moderate intensity, improving the quality of the diet (to the median in the gender and age group), as well as reducing BMI to 25 kg/m^2^ would result in an estimated LE gain of 5.6 years.

The 30-year-old patient B (female) had a normal BMI and median diet quality, but was a cigarette smoker (1 pack per day for 10 years), and her physical activity was low (0 min moderate-to-vigorous leisure-time physical activity). Her estimated LE was 44.8 years (95% UI 44.0–45.6) and was 6.7 years shorter than the population LE in the gender and age group and 20.6 years shorter than the 90th percentile for this group. For such individuals, increasing leisure-time physical activity per week to 150 min of moderate-intensity and quitting smoking would result in an estimated LE gain of 6.7 years.

In the case of patients A and B, a single-factor change was associated with a prolongation of LE by approximately 2.4–4.0 years. However, lifestyle modifications often involve changes in more than one risk factor. When we consider people with all lifestyle characteristics in the same position in the ranking of their gender and age groups, the difference in LE is considerable. For example, the difference between LE for 30-year-old people with risk factors ranked in 90% and 10% of their gender and age groups was 23 years for males and 18 years for females, whereas in 75% and 25%, it was 14 years for males and 10 years for females. Similarly, on comparing 50-year-old people with different lifestyles, the difference between LE for individuals with risk factors ranked in 90% and 10% of their gender and age groups was 24 years for males and 21 years for females, whereas in 75% and 25%, it was 14 years for males and 9 years for females.

## Discussion

### Principal findings

We built a model to determine the effect of lifestyle changes on future LE in young and middle-aged adults. We evaluated the simultaneous influence of lifetime smoking exposure, diet quality, physical activity, and BMI on LE, while maintaining the continuous nature of these predictors. This approach allows for a more accurate assessment of the impact of lifestyle on LE and can also be used to demonstrate the potential risks and benefits of complex lifestyle modifications for educational purposes or to increase motivation to change behavior.

To determine the impact of lifestyle-related risk factors on LE, the period life table for the population was adjusted for the risk factors identified using the logistic risk model. The advantage of this approach is that the presented LE calculation methodology can be easily adjusted for a different target population, and the calculations can be simplified under the following assumptions: (1) populations differ mainly in the absolute risk of death and the prevalence of risk factors, and (2) the relative effect of a health risk factor is similar across populations. In this case, (1) should be determined for a given population, whereas (2) can be adopted from the results of studies conducted on another population.

The complex sampling used in the NHANES and our efforts to preserve the sample structure during model estimation and validation enabled generalization of the study results to the general population.

Our study highlights the fundamental role of healthy lifestyle in preventing premature mortality. For example, the estimated LE at the age of 30 years for individuals with a very-low-risk lifestyle profile (Fig 2, all lifestyle characteristics at 90% in the ranking) was, on average, 18 years longer in women and 24 years longer in men than in those with a very-high-risk profile (Fig 2, all lifestyle characteristics at 10% in the ranking).

In the presented patient examples, the modification of a single lifestyle factor was associated with an LE gain that may be perceived as relatively small compared to the effort required to change habits. Such disparities in LE changes associated with the modification of one versus multiple lifestyle risk factors suggest an intra-individual clustering of these factors. It also points out that health-promoting behavioral changes should address multiple lifestyle areas.

### Comparison with existing studies

To our knowledge, this is the first study to comprehensively quantify the effects of lifestyle on longevity in young and middle-aged adults. Most available research has focused on individuals aged ≥50 years [6–9]. The measures of the risk of death and their changes due to the modification of risk factors are expected to be very low in young people, even with poor lifestyle choices. Only two studies included the younger age group (≥30 years), but both used data from the same cohort to estimate hazard ratios [5, 10]. However, LE was calculated only for participants aged ≥50 years in the first study. By including young adults in our model and using the life table method for LE calculation, we can consider the risk factors that may affect health later in life. The application of this model to the young adult population allows for the early identification of those at risk of premature mortality. This identification can be based solely on behavioral risk factors, even in the absence of classic risk factors such as obesity, diabetes, or dyslipidemia.

Most existing studies on the effects of lifestyle factors on LE utilize data from cohort studies [5–10]. Cohort studies, particularly those on mortality, often require decades of follow-up, during which living conditions and risk of death may change significantly. In a study by Li et al., the follow-up ranged up to 34 years [5]. In this study, we used a cross-sectional approach instead of a longitudinal survey of an actual cohort. This was possible by creating a hypothetical cohort, a technique commonly used in demography but rarely in medical studies. The hypothetical cohort allowed the calculation of LE under the assumption that the mortality pattern observed across age groups during the cross-sectional survey persisted throughout the subsequent life of the hypothetical cohort [15]. Compared to longitudinal data, cross-sectional data are widely available from health surveys routinely conducted in various parts of the world and over various time periods. The presented approach may reduce the sample size requirements, shorten the follow-up duration to reach the advanced age of the study participants, and collect an appropriate number of outcomes (i.e., deaths).

Another advantage of the cross-sectional approach is that the assumption of constancy of the characteristics of study participants is easier to meet within a short period. The levels of risk factors can change over a person’s lifetime, especially in the long term, which is an issue for many long-term cohort studies. Our logistic regression model estimated the risk of death within 2 years of exposure (risk factors). In addition, the life table method of LE calculation allowed us to consider changes in risk factors related to aging and lifestyle changes in the subsequent years of an individual’s life.

Consistent with previous study findings, we found that each additional favorable risk factor was associated with a reduction in all-cause mortality risk [9, 10] and an increase in LE [5, 7, 8]. A cohort study on German and Scandinavian men and women aged ≥50 years found that LE for 50-year-old people with a favorable lifestyle (overweight but not obese, light/moderate drinker, non-smoker, and participates in vigorous physical activity) was 7–16 years longer than for those with an unfavorable lifestyle (overweight but not obese, light/moderate drinker, smoker, and does not participate in physical activity) [8]. In that study, non-smoking yielded the greatest extra years in the overall LE (4–8 years) compared to the effects of the remaining factors. In our study, the effect of smoking on LE was smaller. This may be due to the difference in predictors included in the final model; in study [8], smoking may have taken over the combined effect of unaccounted factors that differed between smokers and nonsmokers. They also assessed smoking exposure in three categories (never, former, and current), in contrast to the continuous variable used in our study, which allowed for a more complete quantification of this exposure.

A study on a US population (aged ≥50 years) found that for 50-year-old men and women, the LE differences between the categories with the best and worst lifestyle profiles were 11 and 12 years, respectively [7]. In that study, dichotomized BMI, smoking, and alcohol consumption were included as behavioral risk factors. The authors of [7] deliberately excluded physical activity from the predictors because it represents both the cause and effect of health status (the possibility of reverse causation), possibly leading to unreliable estimates. In our study, we faced the same issue; however, to preserve physical activity, we decided to pre-specify its effect size based on a literature review [27, 28].

Another study on the impact of lifestyle factors on LE in the US population was conducted by Li et al. [5]. These included smoking status, BMI, physical activity, alcohol consumption, and diet quality, each expressed on a 5-tier scale. The methodology used in that study was similar to that used in our study. The effect on mortality was estimated by combining data from cohort studies (hazard ratios) and a cross-sectional survey (NHANES, distribution of risk factors), and LE was calculated using the life table method. According to their findings, men and women with favorable lifestyle profiles had the projected LE at age 50 on average 14.0 years and 12.2 years longer than those with high-risk profiles, respectively. However, they used data from healthcare worker cohorts and did not account for comorbidities, which may have limited the generalizability of their study. They also used a composite lifestyle score as a predictor, whereas in our model, each lifestyle domain was represented by a separate continuous predictor which limited the bias incurred by information loss.

To refer to the above-presented results from the literature, if we compare 50-year-old individuals in our study, the difference between LE for individuals with low- and high-risk lifestyle profiles is closer to the above literature results for individuals with characteristics at 75% and 25% in the ranking (LE differences: 14 years for males and 9 years for females) than at 90% and 10% in the ranking (LE differences: 24 years for males and 21 years for females).

### Study limitations

The cross-sectional NHANES study employed a range of strategies to mitigate potential biases, including but not limited to random sampling, complex sampling design, standardized protocols, physical examinations, computer-assisted interviews, and data quality control measures. To maintain the accuracy of estimates in our study, we ensured the preservation of the complex sample structures, pooled data from multiple editions, created synthetic features from various aspects of a given factor (e.g. diet quality score), and avoided categorization of continuous variables. To address confounding and the reverse causality bias, we employed a rigorous approach to model development and parameterization. However, these measures could not entirely eliminate all sources of biases, especially self-reporting biases related to participants’ lifestyle and disease history, e.g. responder bias, recall bias, or social desirability bias.

Second, not all the factors that could affect LE were included in our study. In the development of the model, the choice of variables was consciously guided by the purpose of the research, the available number of recorded events (deaths), and the interrelationships between potential predictors. Alternative sets of non-confounding risk factors could be applied depending on the intended use of the model.

During the study, we made the following major assumptions: that covariates for the study participants remained time-invariant over the two-year follow-up period, that the modeled diseases are incurable, and that the mortality pattern observed in the cross-sectional survey would persist throughout the subsequent life of the hypothetical cohort (i.e. period mortality pattern was assumed, rather than the historical mortality pattern of the real cohort).

Finally, our model successfully passed a thorough internal validation using bootstrap resamples of the analyzed data set. However, our future research plans include the more stringent external validation. This could be accomplished by utilizing data from subsequent NHANES editions or datasets from similar cross-sectional surveys.

## Conclusions

In summary, using a cross-sectional approach, we created an LE model that allowed a comprehensive assessment of the effect of lifestyle on mortality in young and middle-aged adults. We found that LE estimates based on continuous predictors representing physical activity, diet quality, smoking history, and BMI showed considerable variability among individuals with different lifestyles. Thus, our model can be applied to the identification of young individuals at risk of premature mortality from noncommunicable diseases and the early implementation of preventive measures.

The results of our research confirmed that lifestyle is a key determinant of lifespan. Premature mortality can be effectively prevented by changing daily habits, and the benefits also extend to healthy, young individuals.

Therefore, a comprehensive lifestyle assessment should be a routine part of clinical evaluation. Our study demonstrates that a more complex and personalised assessment of LE is possible, taking into account its multifaceted aspects, such as the quality of nutrition and physical activity.

Leveraging the potential of mobile technologies and the internet can significantly support the creation and dissemination of a tool that facilitates such assessments, both for individuals and healthcare workers. Moreover, such a tool would allow patients and caregivers to track progress and provide feedback on the health effects of the introduced changes, which can greatly assist in the challenging task of permanently modifying habits.
